# The use of standardized patients for mock oral board exams in neurology: a pilot study

**DOI:** 10.1186/1472-6920-6-22

**Published:** 2006-04-25

**Authors:** Brett Kissela, Steven Harris, Dawn Kleindorfer, Christopher Lindsell, Robert Pascuzzi, Daniel Woo, Jerzy Szaflarski, Daniel Kanter, Alex Schneider, Michael Sostok, Joseph Broderick

**Affiliations:** 1 Department of Neurology, University of Cincinnati, 231 Albert Sabin Way, Cincinnati, OH 45267-0525, USA; 2 Standardized Patient Program, SUNY Upstate Medical University, 2263 Weiskotten Hall, 750 E. Adams Street, Syracuse, NY 13210, USA; 3 Institute for Study of Health, University of Cincinnati, 275 Hastings L. and William A. French Building, 3202 Eden Avenue, Cincinnati, OH 45219, USA; 4 Department of Neurology, Indiana University, 125 Emerson Hall, 545 Barnhill Drive, Indianapolis, IN 46202, USA; 5 Department of Neurology, Mission Hospital, 509 Biltmore Avenue, Asheville, NC 28801, USA; 6 Department of Internal Medicine, University of Cincinnati, 231 Albert Sabin Way, Cincinnati, OH 45267-0557, USA

## Abstract

**Background:**

Mock oral board exams, fashioned after the live patient hour of the American Board of Psychiatry and Neurology exam, are commonly part of resident assessment during residency training. Exams using real patients selected from clinics or hospitals are not standardized and do not allow comparisons of resident performance across the residency program. We sought to create a standardized patient mock oral board exam that would allow comparison of residents' clinical performance.

**Methods:**

Three cases were created and then used for this mock oral boards exercise utilizing trained standardized patients. Residents from the University of Cincinnati and Indiana University participated in the exam. Residents were scored by attending physician examiners who directly observed the encounter with the standardized patient. The standardized patient also assessed each resident. A post-test survey was administered to ascertain participant's satisfaction with the examination process.

**Results:**

Resident scores were grouped within one standard deviation of the mean, with the exception of one resident who was also subjectively felt to "fail" the exam. In exams with two faculty "evaluators", scores were highly correlated. The survey showed satisfaction with the examination process in general.

**Conclusion:**

Standardized patients can be used for mock oral boards in the live patient format. Our initial experience with this examination process was positive. Further testing is needed to determine if this examination format is more reliable and valid than traditional methods of assessing resident competency.

## Background

Currently, the American Board of Psychiatry and Neurology (ABPN) utilizes a live patient hour as one form of assessing candidates during the Step Two examination. This includes thirty minutes for the candidate to obtain a history and perform a physical examination on a patient with a neurologic disorder, and is followed by questioning in which candidates explain their thought process for evaluating the patient. Concerns have been raised about the reliability and validity of this exam, since the experience is not standardized. For example, some patients may openly reveal their diagnosis, or the diagnosis may be more readily evident than for other patients. For these reasons, the ABPN plans to replace the patient hour with other forms of assessment by 2008.[1]

Residency programs commonly test their residents in "mock oral board exercises" that simulate the ABPN patient hour, using actual patients with a neurologic disease. Residents benefit from this formative assessment in that they can become familiar with the ABPN format prior to their actual boards exam, and can obtain valuable feedback about their performance in a observed clinical encounter. Faculty can evaluate the resident's history-taking and interpersonal communication skills, physical exam skills, and thought process regarding patient management; and can thus evaluate multiple ACGME Core Competencies (Patient Care, Medical Knowledge, Interpersonal and Communication Skills, and Professionalism) making these mock examinations useful resident evaluation tools for Program Directors who must demonstrate resident competence in these areas prior to graduation. However, to perform "mock oral boards" using real patients in the outpatient or hospital settings, the same logistic considerations apply as for the ABPN exam. Each resident assessment involves a different patient and is examined by different supervisory physicians, so that assessment is not standardized and no comparisons can be drawn between residents.

Some of the disadvantages of both the ABPN patient hour and "mock oral boards" during residency training could be reduced by using standardized patients (SPs), or professional actors well-versed in simulating neurologic disease. SPs allow the exam to be standardized for each individual, allow comparisons across individuals tested with the same SP, and allow uniform testing over time. The SP can also evaluate the residents' performance in the context of a standardized assessment, providing feedback from the patient's perspective.

Although SPs were first used more than 30 years ago to teach and test medical students' clinical skills, reliance on the SP interaction as a teaching and testing tool has increased greatly in medical schools [2-15], as well as in residency programs across many disciplines [14-23]. The importance of the SP interaction in medical education is demonstrated by the addition of an OSCE known as the USMLE Step 2 Clinical Skills (CS) examination as a new requirement for medical licensure in 2004. Reports in the literature confirm that short SP encounters in objective structured clinical examinations (OSCE) are a valid and reproducible testing, and much work has been done to validate the use of SP exams across many disciplines of medical training. However, the use of SP's for neurology mock oral board exams is a novel and innovative application, particularly as it is unknown whether SPs can accurately portray a patient with neurologic problems.

We describe our initial experience with utilizing standardized patients in a "mock oral board" format. Our broad objective was to determine if this exercise was a practical and useful alternative to utilizing actual patients for mock oral boards. We specifically wanted to determine if SP's could successfully portray neurologic patients to the satisfaction of the residents and faculty involved in the exercise.

## Methods

We utilized resources and personnel from the Center for Competency Development and Assessment (CCDA) at UC to develop three SP cases used for our mock oral exam. These three cases were adapted from patients seen in clinical practice at the UC. Cases were designed with an increasing level of complexity for each level of training. For example, the PGY-2 case was a straightforward case of a right hemisphere stroke (obvious clinical findings), the PGY-3 case was a frontal brain tumor who had presented with a seizure (subtle exam findings), and the PGY-4 case was the most complex with a diagnosis of Devic's disease (complicated history and exam findings indicating multiple lesions in the central nervous system). The final case "scenarios" are included as Appendices 1–3. [see Additional file 1] [see Additional file 2] [see Additional file 3] A global fee was assessed for the utilization of the CCDA facilities and staff time.

Each case was assigned to one of three SPs contracted with the University of Cincinnati College of Medicine for CCDA activities. Each had volunteered for SP duties previously, and 3 of the best performers were selected for this exercise. They were paid on an hourly basis commensurate with CCDA policy. None of the SP participants had a pre-existing neurologic diagnosis.

Each individual SP met with two of the authors (BK, SH) for four training sessions of 1–1.5 hours each, in which the history and exam findings for their case were first taught and then practiced. Videos of physical findings, (such as hemiparesis, spasticity, deep tendon reflex hyper or hypoactivity, visuospatial neglect, facial droop, and appropriate emotional affect) taken from CD-ROM textbooks where available were used as examples to help the SP understand the physical findings to be simulated. Each SP finally underwent a "dress rehearsal" in which another faculty neurologist (DK or AS) examined them and provided feedback on performance; no retraining was considered necessary. All neurology faculty donated their time.

Each resident was asked to sign an Honor Code Agreement prior to the examination, in which they promised not to reveal any details of the exercise to their colleagues either before or after their exam. In this way, cases can be reused in future years.

The mock oral board exercises for UC residents (n = 3 for PGY-2, n = 3 for PGY-3, and n = 2 for PGY-4) were performed at the UC Center for Competency Development and Assessment (CCDA) on June 19, 2002. Three PGY-4 residents from Indiana University were later tested on October 2, 2002, increasing the number of PGY-4 residents tested to five. The exercise was required for trainees at the University of Cincinnati, taking the place of the yearly "mock oral boards". The exercise was voluntary for trainees from Indiana University. Both exam sessions were conducted in one afternoon each, where each of the SPs was examined by 2 or 3 residents from the given PGY level of training specific to their case. All resident cases will hereafter be described by level of training.

Each resident was directly observed by one or two neurology faculty who were present in the room in a manner similar to the ABPN examination. Each resident was scored in a standardized fashion, using an assessment form similar to those used by the ABPN for scoring the live patient hour. Each part of the history and examination is listed on the form, and the resident's performance on each was numerically graded as unsatisfactory (1), borderline (2), or satisfactory (3). Additional general areas of assessment, using the same 1–3 scale included communication skills, respect for the patient, and concern for patient safety. For assessment forms completed by faculty, the maximum possible score was 75 regardless of the year of training. A space was also provided for note-taking and for faculty to provide written comments. The attending physicians were asked to make a subjective final pass/fail determination for each resident exam, using criteria similar to the ABPN examination. A sample assessment sheet is included as Appendix 4 [see Additional file 4].

The standardized patient was given an assessment sheet with case-specific details regarding the history and examination. The SP marked the sheet if the resident obtained the relevant history or exam component. The maximum possible scores varied by case due to differing complexities in history or exam. The SP was given explicit details regarding performance of each part of the history and exam, but was allowed to divulge historical information in a way that felt natural to them. The SP answered several yes/no questions at the end of the assessment form, such as "Did the resident make you feel comfortable" and "would you be comfortable seeing this doctor again". The SP assessment sheet for each case is included with the case scenario in Appendices 1–3. [see Additional file 1] [see Additional file 2] [see Additional file 3]

The PGY-3 case with a brain tumor had an additional twist. It was expected that the resident would ultimately consider brain tumor as a potential cause for the patient's symptoms, and would consider brain imaging as part of the patient's workup. After the discussion of the resident's thought process regarding the case had been completed, the resident was informed that imaging studies had been performed and a brain tumor had been found. The resident was then asked to go back into the room and "break the bad news" to the SP. This is not in keeping with ABPN exam format, but provides for assessment of the resident in end of life care issues. Specific instructions were given to the SP for this portion of the encounter (see Appendix 2) [see Additional file 1]. A separate faculty assessment form and SP checklist was provided for this exercise (see Appendix 5). [see Additional file 5]

After all residents had completed the exam, faculty members and residents were asked to complete a survey about their experience. Not all participants completed this survey, although those who did so answered every question. Each survey question utilized a 1–5 Likert scale (1 = strongly agree, 5 = strongly disagree).

### Statistical Analyses

This analysis of data gathered for program and resident evaluation was classified as exempt from review by the Institutional Review Board under category 1 (research on normal education practices) and category 2 (research involving educational tests and survey procedures), as defined in 45 CFR 46.101(b). Summed scores from each physician and SP were calculated for each resident. For residents who had two examiners, inter-observer agreement for summed scores was evaluated using the mean difference in summed scores, the correlation between summed scores using Pearson's correlation coefficient, and Lin's concordance correlation coefficient. The mean of the two scores was used as the score for that resident for that SP. Within each year of training, a mean and standard deviation score for all residents was calculated to measure performance on the test.

For each question on the survey, mean responses were calculated for residents and attendings independently. Differences between faculty and residents on each survey item were tested using the student's t-test. The t-test is relatively robust to non-normality and comparisons were checked using non-parametric statistics (Mann-Whitney U test); t-tests are reported here to be consistent with reporting of means and standard deviations.

## Results

As described above, eleven residents participated in this exercise (3 from PGY-2, 3 from PGY-3, and 5 from PGY-4). Five UC neurology faculty members, one IU neurology faculty member, and one educator from UC participated in scoring the SP exercise. One UC neurology faculty (AS) participated in training SPs but not in scoring the exams.

Mean attending physician scores (+/- standard deviation) by year of training are presented in Table 1. Only one resident fell greater than one standard deviation from the mean score for his/her level of training. This resident was the only resident subjectively determined by attending examiners to have "failed" this exercise. Only 6 residents were examined by two faculty simultaneously (due to problems in scheduling faculty for an extended block of time). Summed scores differed by 0, 1, 1, 1, 4, and 5 points respectively (mean difference = 2.0; Pearson Correlation Coefficient = 0.99, Lin's concordance correlation coefficient = 0.5352)

Common areas of "unsatisfactory" performance on faculty evaluations included not washing hands prior to patient exam (8 of 11 did not wash hands) and not taking vital signs (9 of 11 did not take vital signs). Four residents did not ask for the patient's age, three residents did not ask for the patient's handedness, and three residents did not ask for a history of allergies to medications. In the real ABPN exam these items are often neglected due to the time constraints.

SP scores and possible points are presented in Table 2. As described above, SPs had only yes/no checklist scoring sheets and the number of evaluation points varied by case. SP checklist evaluations were less discriminating, in that no resident scored outside the standard deviation.

Tables 1 and 2 also show the scores for the "breaking the bad news exercise" performed by the PGY-3 residents. While this was an artificial situation, it was taken seriously by the residents. All 3 residents were given immediate feedback from the SP and the examiners at the end of this exercise. No systematic errors were made by the residents, although several suggestions were made with regard to style and mannerisms. All 3 resident participants felt that this was a useful exercise that helped them with their skill in breaking bad news to a patient. All three also felt that this part of the exam should be repeated in subsequent years (verbal communication).

Results of the post-exercise survey are presented in Table 3. The table shows that both residents and faculty found this exercise to be a useful way of assessing the resident's skills. One of the biggest concerns voiced by faculty prior to the exam was whether the SPs would be able to stay in character consistently for the entire 30 minutes and reliably reproduce examination findings. The survey suggests shows that both faculty and residents thought the simulation was realistic.

There were no significant differences found in answers from residents and faculty on duplicated survey questions, although there were modest trends towards differences in opinion with regard to the effectiveness of assessing resident skill in patient care and the value of videotaping the encounters. The faculty thought that videotaping the encounters for review would be a valuable training tool, while residents were less enthusiastic. It should be noted that these encounters were not videotaped, although the CCDA has the capability of doing so.

## Discussion

Mock oral boards are a useful way of assessing resident performance during training, since multiple ACGME Core Competencies can be evaluated simultaneously, including issues surrounding end of life care. Standardized patients are used for medical student education, but the use of SP's for a mock oral board examination is a novel variation that offers several advantages. Each level of training can be tested in the same way on the same patient, to allow direct comparison between residents at each level. These cases will be used again each year, and can be used to compare resident performance over time. Maintenance of the same score on faculty evaluations would be demonstration of growing competence over time, as the next year of training's case is more complicated and requires demonstration of a higher level of competence as a neurologist. As we accumulate experience with this testing, we hope to demonstrate the reliability and validity of this exercise. If we find that this exercise is reliable and valid then we could use this exercise as an outcome measure for competency assessment as will be required by the ACGME. Results on this exercise can be compared to USMLE scores, monthly evaluation results from faculty and peers, and ultimately with success on the ABPN Board Examinations to further determine validity.

The data presented are our initial pilot experience with the SP exams. Given the small numbers of residents who participated, few conclusions can be drawn. Our aim was to report the innovative methodology, and to determine if the data support continuation of this practice. We are encouraged by the agreement between examiners for residents where multiple faculty members could participate. We are further encouraged that the one resident whose score fell more than one SD from the mean was also the only resident determined to subjectively "fail" the exam. However, the reader must interpret the results with caution given the small sample tested with each case. More data will be needed before we can determine if our exam is reliable and/or valid.

The survey shows that residents and faculty alike found this exercise to be a valuable experience. One of the biggest concerns prior to the exam was the ability of the SP to stay in character consistently for the entire 30 minutes, and to reliably reproduce examination findings. The results of the survey show that our SPs were indeed able to do so without difficulty, as both faculty and residents felt that the exam was realistic. Furthermore, the faculty felt that the SPs were able to consistently deliver the same information and portray the same physical findings, suggesting that differences in resident score are not caused by variability in SP performance. In the future, we plan to implement videotaping of these encounters, and will assess the usefulness of reviewing the video with the residents as part of the feedback. Alternatively, we may ask the resident to review their taped encounter and score themselves as part of an annual self-assessment exercise. Finally, we intend to add case-specific imaging studies for interpretation by the resident as part of the exercise, so that we can test competency in interpretation of neuroimaging studies.

A major weakness of this pilot study was the inability to have 2 faculty evaluators for each resident tested, further limiting conclusions that can be drawn from the results. For example, examiner variability cannot be examined sufficiently with our pilot data. Obtaining 2 faculty evaluators was a logistic challenge that may limit the ability of some programs to carry out similar exercises. The inability to obtain 2 evaluators in this case was due to the corresponding author's inexperience with organizing an exercise of this type. This has been improved with better planning in subsequent years.

## Conclusion

Standardized patients can be used for mock oral boards in the live patient format. Our initial experience with this examination process was positive. We will continue to administer this SP mock oral boards exercise to our residents and track our results, with the ultimate goal of testing the reliability and validity of this exercise. We will use the results to assess resident competency and to look for systematic weaknesses in our residency training program.

As the American Board of Psychiatry and Neurology replaces the current form of the live patient exam, they will consider other ways of testing clinical competency in residency graduates. Our initial experience with standardized patients suggests that the use of SPs may be one option for the Board to consider.

## Competing interests

The author(s) declare that they have no competing interests.

## Authors' contributions

BK and SH conceived of the study, all authors participated in the design and coordination of the study, and CL performed the statistical analysis. All authors read and approved the final manuscript.

## Pre-publication history

The pre-publication history for this paper can be accessed here:



## Supplementary Material

Additional file 1 Appendix 1-Case Scenario for "Dorothy McMillan" (PGY 2) Click here for file

Additional file 2 Appendix 2-Case Scenario for "Peggy Cusick" (PGY 3) Click here for file

Additional file 3 Appendix 3-Case Scenario for "Phyllis Jones" (PGY 4) Click here for file

Additional file 4 Appendix 4-Faculty Evaluation Form Click here for file

Additional file 5 Appendix 5-Breaking Bad News Exercise-"Peggy Cusick" SP Checklist Click here for file

## Figures and Tables

**Table 1:** Attending Evaluation Scores By PGY-level

	n	Maximum score possible	Mean	SD	range
PGY-2	3	75	68.4	4.6	63–73
PGY-3	3	75	68.2	1.9	65–70
PGY-3 ("bad news")*	3	27	22.0	2.6	20–27
PGY-4	5	75	68.9	3.9	61.5–73

*"bad news" = exercise in breaking the bad news to the patient that a brain tumor had been discovered on neuroimaging

**Table 2:** Standardized Patient Evaluation Scores

	n	Maximum score possible	Mean	SD	range
PGY-2	3	21	17.7	0.5	17–18
PGY-3	3	27	24.7	0.5	24–25
PGY-3 ("bad news")*	3	6	5.3	0.5	5–6
PGY-4	5	28	22	1.6	20–24

*"bad news" = exercise in breaking the bad news to the patient that a brain tumor had been discovered on neuroimaging

**Table 3:** Mean Scores (+/- SD) on Post-Examination Survey

Item	Faculty (n = 4)	Residents (n = 8)
The SP history was realistic.	1.5 (0.6)	2.3 (1.3)
The patient simulation of physical exam findings was realistic.	2.4 (0.5)	1.8 (0.7)
The SP exercise effectively evaluated communication skills.	1.3 (0.5)	1.5 (1.1)
The SP exercise effectively evaluated physical exam skill.	1.5 (0.6)	1.5 (0.8)
The SP exercise is a valid way to assess the resident's knowledge.	1.8 (0.5)	1.5 (0.8)
The SP exercise is a valid way to evaluate resident's professionalism.	1.8 (1.0)	2.5 (1.7)
The SP exercise is a valid way to assess resident's skill in patient care.	2.3 (0.5)	1.5 (0.8)^#^
The SP exercise is a valid way to access resident's safety in patient care.	1.3 (0.5)	1.5 (0.8)
Video taping this exercise would be a useful feedback tool.	1.3 (0.5)	2.4 (1.5)^##^
This was an excellent simulation of the ABPN live patient exam	2.0 (0)	
The SPs were consistent in their delivery of the history.*	1.33 (0.6)	
The SPs were consistent in their portrayal of exam findings.*	1.3 (0.6)	
The resident was distracted by inaccurate portrayal of findings.	3.5 (0.6)	
This exercise was useful for me.		1.1 (0.4)

Evaluation using Likert Scale: (1 = Strongly Agree; 2 = Agree; 3 = Neutral; 4 = Disagree; and 5 = Strongly Disagree)

* only answered by faculty who observed the same SP in more than one resident encounter

•^# ^p = 0.07

•^## ^p = 0.09

## References

[B1] (2003). Personal communication to Consortium of Neurology Program Directors by Stephen Scheiber, Executive Vice President, American Board of Psychiatry and Neurology, at the American Academy of Neurology's 55^th ^Annual Meeting in Honolulu, HI.

[B2] Blake KD, Mann KV, Kaufman DM (2001). Using standardized patients to identify students needing extra training in interviewing skills. Acad Med.

[B3] Brazeau C, Boyd L, Crosson J (2002). Changing an existing OSCE to a teaching tool: the making of a teaching OSCE. Acad Med.

[B4] Colliver JA, Swartz MH, Robbs RS, Lofquist M, Cohen D, Verhulst SJ (1998). The effect of using multiple standardized patients on the inter-case reliability of a large-scale standardized-patient examination administered over an extended testing period. Acad Med.

[B5] Makoul G, Altman M (2002). Early assessment of medical students' clinical skills. Acad Med.

[B6] McGraw RC, O'Connor HM (1999). Standardized patients in the early acquisition of clinical skills. Med Educ.

[B7] Rose M, Wilkerson L (2001). Widening the lens on standardized patient assessment: what the encounter can reveal about the development of clinical competence. Acad Med.

[B8] Solomon DJ, Szauter K, Rosebraugh CJ, Callaway MR (2000). Global Ratings of Student Performance in a Standardized Patient Examination: Is the Whole More than the Sum of the Parts?. Adv Health Sci Educ Theory Pract.

[B9] Towle A, Hoffman J (2002). An advanced communication skills course for fourth-year, post-clerkship students. Acad Med.

[B10] Barrows HS (1993). An overview of the uses of standardized patients for teaching and evaluating clinical skills. AAMC Acad Med.

[B11] Vu NV, Barrows HS, Marcy ML, Verhulst SJ, Colliver JA, Travis T (1992). Six years of comprehensive, clinical, performance-based assessment using standardized patients at the Southern Illinois University School of Medicine. Acad Med.

[B12] Williams RG, Barrows HS, Vu NV, Verhulst SJ, Colliver JA, Marcy M (1987). Direct, standardized assessment of clinical competence. Med Educ.

[B13] Hodges B, Regehr G, Hanson M, McNaughton N (1997). An objective structured clinical exam for evaluating psychiatry clinical clerks. Acad Med.

[B14] Nagoshi M, Williams S, Kasuya R, Sakai D, Masaki K, Blanchette PL (2004). Using Standardized Patients to Assess the Geriatrics Medicine Skills of Medical Students, Internal Medicine Residents, and Geriatrics Medicine Fellows. Acad Med.

[B15] Hodges B, Regehr G, Hanson M, McNaughton N (1998). Validation of an objective structured clinical exam in psychiatry. Acad Med.

[B16] Beeson MS, Wilkinson LF (1999). Evaluation of clinical performances of emergency medicine residents using standardized patients. Acad Med.

[B17] Donnelly MB, Sloan D, Plymale M, Schwartz R (2000). Assessment of residents' interpersonal skills by faculty proctors and standardized patients: a psychometric analysis. Acad Med.

[B18] Duke MB, Griffith CH, Haist SA, Wilson JF (2001). A clinical performance exercise for medicine–pediatrics residents emphasizing complex psychosocial skills. Acad Med.

[B19] Norcini JJ, Blank LL, Duffy FD, Fortna GS (2003). The mini-CEX: a method for assessing clinical skills. Ann Intern Med.

[B20] Regehr G, Freeman R, Robb A, Missiha N, Heisey R (1999). OSCE performance evaluations made by standardized patients: comparing checklist and global rating scores. Acad Med.

[B21] Roth CS, Watson KV, Harris IB (2002). A communication assessment and skill-building exercise (CASE) for first-year residents. Acad Med.

[B22] Serwint JR (2002). The use of standardized patients in pediatric residency training in palliative care: anatomy of a standardized patient case scenario. J Palliat Med.

[B23] Sibert L, Grand'Maison P, Doucet J, Weber J, Grise P (2000). Initial experience of an objective structured clinical examination in evaluating urology residents. Eur Urol.

