# Supplementary material for: The use of standardized patients for mock oral board exams in neurology: a pilot study
Source: BMC Med Educ. 2006 Apr 25;6:22. doi: 10.1186/1472-6920-6-22 (PMC1464094; doi:10.1186/1472-6920-6-22)
Supplement: Additional file 1 — Appendix 1-Case Scenario for "Dorothy McMillan" (PGY 2) [file 1472-6920-6-22-S1.doc]

**Appendix 1—Case Scenario for “Dorothy McMillan” (PGY 2)**

**Case Summary:**

You are a 64-year old woman who has come to the emergency room today because your left face, arm and leg feel weak and numb. The left side of your face droops slightly and your speech is slightly slurred.

# History of Your Weakness

When you woke up from my daily nap, you had difficulty when you tried to get up from the bed to go to the bathroom. You called your husband from the other room and he decided to bring you to the doctor. He was very concerned that you had a stroke. He said that the left side of your face drooped and your speech was slurred. He says that your left arm and leg aren’t working right. You haven’t noticed any of this; in fact you insist that you feel fine. You are neglecting or ignoring the entire left side of the world for the most part. You are NOT completely unaware of your left side; but you do not complain of anything being wrong.

You do not use your left arm or leg during the encounter. You do have a facial droop on the left side, which causes your speech to slur. You tend to look to your right side only, and see things only to your right or directly in front of you. If the doctor moves to your left side; you turn your entire head to the right to keep him/her in your field of vision.

If asked about previous similar episodes, you tell about three days ago when you had a sudden weakness in your left arm. If asked, you didn’t notice any numbness. You arm just felt “heavy” and didn’t seem to be working right. It went away on its own after an hour or so. You mentioned it to your husband, but did not seek medical attention. You have not had other similar episodes.

Your husband is not in the room with you because he has gone to call your daughter who lives in the area.

# Past Medical History

You have asthma. You have had it for “years”. You have an inhaler (albuterol) at home for emergencies, but haven’t needed it in over a year.

8 years ago you had a “total hysterectomy” because you had cervical cancer. You did not have chemotherapy or radiation after the surgery. You have had no problems since.

3 months ago you had a case of “the shingles”. You suddenly broke out in a blistery rash around your right eye. It was very painful and you gradually lost your vision in that eye until it was almost gone.

The doctor put you on acyclovir (10 days) for the shingles. Your vision gradually returned over the next week, but you’re not sure it ever got back to normal. The rash also gradually went away over several days. While taking the acyclovir you developed mild kidney problems. Your liver and kidneys seem to be ok now.

You are currently (last 6 months) undergoing chemotherapy with fludarabine for a form of leukemia; they call it CLL. Your liver and spleen were enlarged which led to them diagnosing the CLL. You had monthly IV treatments. You can’t remember how many, but the last one was last month.

You are currently taking Prednisone ( a steroid) as part of your chemo. You started out taking 40 mg a day and they have tapered the dosage over the last few months down to 10 mg a day. You also have prescriptions for Oxycontin and Roxicet for pain. You haven’t had to take much of either of the pain meds.

You have no allergies that you know of.

# Family History

Your mother died of cervical cancer about three years ago.

You father and his brother had coronary artery disease (“hardening of the arteries”). Your father died from his heart attack at age 72. Your uncle died of heart failure several months after his heart attack. He was 66.

# Social History

You are retired about 3 years ago. You were really looking forward to retirement, but it seems like the last couple years have just been one medical problem after another.

You live with your husband of 37 years.

You quit smoking about 5 years ago. Before that you smoked about a ½ pack of cigarettes a day for over thirty years. You have never been much of a drinker and haven’t had any alcohol in at least 10 years. You are surprised if asked about other drugs and report that you have never done any of that junk.

# Review of Systems

If asked directly about the following:

1. You do seem to be “depressed” more lately then usually and have at times found yourself crying without much reason
2. You seem to have lost interest in many of the things that you used to enjoy.
3. You feel very tired lately.
4. You appetite seems to be less lately.

# Physical Exam

During the entire encounter you have a somewhat flat affect. As above, you are not completely aware that something is wrong.

The left side of your mouth droops a little and is slow to move if you smile. You can wrinkle both sides of your forehead if asked to and your ability to hold your eyes closed is equal on both sides. On visual testing, you have no vision to the left of your nose in either eye. Your mouth droops slightly on the left and is slow to move. Your tongue works normally.

You exhibit pronator drift in your left arm. You have weakness in your left arm; which is severe at the hand and wrist, less so in the forearm and mild in the shoulder. You can barely resist force from the examiner in your left hand and have a hard time moving it at all. In your upper arm; you have 60-75% of your normal strength.

Remember that the pattern of weakness is important. You are weak in your face from your eyes and below. You hands and wrist are weak and the weakness lessens as you move up your left arm. Your legs are essentially unaffected.

You have loss of sensation on the left side. You report about 25-50% of the right side when comparing sensation from side to side. If asked to close you eyes and identify touch on your left side, right side or both; you will identify touch correctly on either side, but will only feel it on the right if touched simultaneously on both sides. This is true of your arms only. Your legs are normal.

You have brisk and faster reflex responses on your left than on your right. The size of the movement is not affected; only the speed of the twitch. Ankle reflexes are normal.

Your left big toe goes up if they scratch the bottom of your foot, side of your foot, if they run their knuckles down the front of your leg, or if they stick a pin into the top of your big toe. The upgoing movement should be very subtle and if you have a hard time, it is better for it to not move (often seen) as compared to an exaggerated upward movement (doesn’t look real).

Subtle is better. The last important thing is consistency—the weakness described above must be there at all times!!—during the history and the exam.

Dorothy McMillan SP Checklist

**Resident Name _________________________________________________**

**SP Name ___________________________________________________**

The doctor discovered that:

1. I am 64 years old. ______

2. I am right/left handed. ______

3. I am here because my husband is afraid I may have had a stroke. ______

4. I had a difficult time getting up from a nap earlier today. ______

5. My husband says that I have a facial droop and my speech is slurred. ______

6. I have not noticed anything particularly wrong. ______

7. I had an episode of weakness in my left arm several days ago. ______

8. I recently had a case of “the shingles” which involved loss of my vision. ______

9. I had a total hysterectomy 8 years ago. ______

10. I am currently having chemotherapy for CLL. ______

11. I am taking Prednisone. ______

12. I have no known drug allergies. ______

13. I have a family history of heart disease (father and uncle). ______

14. I quit smoking 5 years ago. ______

15. I drink very rarely. ______

16. I have felt more “depressed” than usual lately. ______

17. I have been very tired lately. ______

18. I understood the doctor clearly during the history and exam. ______

19. The doctor used words that I could understand. ______

20. The doctor treated me with respect. ______

21. The doctor was mindful of my safety during the exam. ______

Comments: (comment specifically on your overall comfort level with the physician; would you come back to him/her)
