# Supplementary material for: The use of standardized patients for mock oral board exams in neurology: a pilot study
Source: BMC Med Educ. 2006 Apr 25;6:22. doi: 10.1186/1472-6920-6-22 (PMC1464094; doi:10.1186/1472-6920-6-22)
Supplement: Additional file 2 — Appendix 2-Case Scenario for "Peggy Cusick" (PGY 3) [file 1472-6920-6-22-S2.doc]

# Appendix 2—Case Scenario for “Peggy Cusick” (PGY 3)

**Case Summary:**

You are a 55-year old woman who has come to the doctor’s office in follow-up after being discharged from the hospital neurology service. You were admitted to the hospital from the ER three days ago after a seizure and fall.

# History of Your Seizure

Three days ago you were walking from your kitchen to the dining room when you suddenly stopped and stared blankly into space for a few seconds. After a few seconds of staring into space, you fell to the floor. Your husband was sitting in the dining room and saw the whole episode. When you did not respond to him, he got scared and called 911. He says that you were unconscious for about 5 minutes, but you have no idea how long it was. The next thing you remember is riding in the ambulance. The ambulance took you to the emergency room. You have no recollection of any of this episode until you were in the ambulance. You only know what your husband has told you. He says that you were awake, but groggy when the ambulance arrived and remained that way all the way to the hospital.

You were not injured in the fall (didn’t hit your head, or bite your tongue etc.). You did not lose control of your bladder or have any jerking movement while you were on the floor (this is what your husband has told you). You do not offer any of this information unless directly asked about it.

At the ER, they did a CT scan and admitted you. After about 2 ½ hours you returned fully to normal. They did not give you any medicine or do any other procedures at that time. They said that you had had a seizure of some sort. They kept you in the hospital overnight for observation. They told you that the CT was “abnormal and they wanted to keep you for a few days to run some tests, but you refused. You preferred to do this on an outpatient basis.

You were referred for this visit in the Neurology Clinic. You had an MRI yesterday afternoon. You are here today to find out the results. You are **very anxious** finding out about the results of the MRI. They told you not to drive a car for 3 months at the ER, so you took a cab to this visit. Your husband had a work engagement that he couldn’t get out of. You really need for the doctor to be reassuring and supportive.

Your husband has noticed the facial droop and slurred speech and mentioned it to you. After he mentioned it; you have noticed that you talk more slowly sometimes. Neither you nor your husband recalls this being present before the seizure.

# Past Medical History

You had by-pass surgery about 6 years ago.

You have had endometriosis over the years. Sometimes it is painful enough to require painkillers (Vicoden). You are on birth control pills to prevent it and they have worked fairly well. You do NOT list them with other medications, but only mention them if asked about the endometriosis. Your endometriosis symptoms include crampy abdominal pain during menstrual cycles with pain radiating into your back. If asked, sexual intercourse was often painful for you.

You sometimes suffer from “reflux” for which you take Pepcid (prescribed). You suffer from seasonal allergies. You take Allegra-D (prescribed).

You have some problems with stress incontinence (urine leakage). You wear a pad in your underwear and try to make frequent and regular trips to the bathroom.

You take a daily vitamin.

# Family History

Your father had lung cancer that metastasized into his brain. You remember him having seizures in which all four of his limbs twitched uncontrollably and he would lose control of his bladder. It was awful.

You have an extensive family history of heart disease on your mother’s side. You have a brother and a sister; both are healthy.

You have no other family history of strokes, seizures, migraines or brain tumors.

# Social History

You work as a supervisor for an advertising agency. You have not been working since your seizure. You have stayed home for the most part because you can’t drive and you are afraid it could happen again. Your husband has his own business and has been very busy the last few days and hasn’t been able to stay with you as much as you’d like.

You’ve been married for 23 years and are quite happy. You understand that he has to take care of business, but you wish he were around more.

You have two children, both of whom are in college out of state. It just you and your husband at home now.

You smoke about 2 packs of cigarettes a day (since your were in your 20’s), but have quit since the seizure. You’ve been trying the nicotine gum, but it’s been very difficult. They told you that your smoking may have contributed to the seizure, so you are determined to quit. You don’t drink any alcohol. If asked about other drugs, you say that you smoked a little pot in college, but that was a long time ago.

# Review of Systems

If asked directly about the following:

1. You are extremely anxious since the seizure; to the point of tears at times.
2. You are very tired, because you’ve had trouble sleeping.
3. You’ve had headaches quite a bit the last few days. If asked, you think they are worse when you lay down, but you can’t be sure.
4. You have NOT had any nausea or vomiting.

All of the ROS symptoms have been around since before the seizure, it’s hard to say exactly how long. They seem to be worse since the seizure.

# Physical Exam

During the entire encounter you sometimes have difficulty finding words. You are slow in answering questions and performing tasks. You occasionally slur your words and have a slight droop on the right side of your face. Remember to keep this consistently—do not forget when asked to open your mouth that the right side of your face should move less. Your facial weakness is from the eye down. If asked to wrinkle your forehead, you can do so normally. Your ability to keep your eyes closed is the same on both sides. Your tongue moves normally.

For strength testing—remember that consistency is the key (your right arm is consistently weak throughout the exam, so you will move it less spontaneously). Do not forget and move it normally during other parts of the exam, such as when leg strength is being tested, or when reflexes are being checked.

The other key point is to minimize, rather than maximize your findings on exam. It does not look correct if you overplay or emphasize your findings. Being abnormal in a subtle way despite “best effort” is what this patient would do.

You have pronator drift on your right side. You have mild weakness in your right arm. Remember to keep about 60-80% power throughout the movement tested—smooth “weakness” without jerks or “rachety” feeling. Be prepared for sudden jerking. The pattern of weakness is important—it will be worse in your hand and wrist than higher in your arm (shoulder, etc). Your legs will be normal.

Sensation is normal—just do your best!

Your reflexes are exaggerated on the right side of your body. You have brisk or faster reflex responses on the right compared to the left. Remember that it is the speed of the muscle twitch that counts and DO NOT exaggerate the movement caused by the reflex (does not look real).

Your right big toe goes up if they scratch the bottom of your foot, side of your foot, if they run their knuckles down the front of your leg, or if they stick a pin into the top of your big toe. The upgoing movement should be very subtle and if you have a hard time, it is better for it to not move (often seen) as compared to an exaggerated upward movement (doesn’t look real).

**BREAKING THE BAD NEWS:**

The doctor will step out and return in about 15 minutes. He will break the bad news to you. Your MRI showed a brain tumor.

You will first be stunned but then will (within minutes) become somewhat “resigned” to the diagnosis. You had suspected it all along, given your father’s history. You should try to be in character and act how you think you would typically act in this situation (i.e. if you want to cry, go ahead—but we would also like you to be consistent for all residents who will examine you on this day if possible).

You should ask the doctor to explain:

1. What this means to you (i.e. will you die from this?) Will you need surgery? Will you need chemotherapy or some other kind of treatment? What else will happen?
2. Is it lung cancer (like Dad’s)?
3. Is the tumor related to the spell I had? Will I have more seizures (like Dad did)?
4. If I will die from this, how will it happen?—will I suffer in any way?
5. Where do we go next?--(help them wrap it up)

# Peggy Cusick SP Checklist

**Resident Name _________________________________________________**

**SP Name ___________________________________________________**

The doctor discovered that:

1. I am 55 years old. ______

2. I am right/left handed. ______

3. I am here following an ER visit and hospital admission. ______

4. I had a “seizure” three days ago. ______

5. My husband says that I suddenly stared blankly into space for several seconds. ______

6. After staring into space I fell down. ______

7. I did NOT injure my self in the fall. ______

8. I did not lose control of my bladder during the seizure. ______

9. I do NOT remember anything about the seizure. ______

10. My husband called an ambulance to take me to the ER. ______

11. After several hours in the ER, I was “fine”. ______

12. A CT Scan was done and I was told that it was “abnormal”. ______

13. I had an MRI yesterday. ______

14. I had bypass surgery 6 years ago. ______

15. I take birth control pills for endometriosis. ______

16. I suffer from seasonal allergies. ______

17. A experience some stress incontinence. ______

18. I have a history of heart disease on my mother’s side. ______

19. My father had seizures resulting from brain tumors. ______

20. I smoke 2 packs of cigarettes a day for the past 30 years. ______

21. I don’t drink alcohol. ______

22. I have had trouble sleeping for several months. ______

23. I have not experienced any nausea or vomiting. ______

24. I understood the doctor clearly during the history and exam. ______

25. The doctor used words that I could understand. ______

26. The doctor treated me with respect. ______

27. The doctor was mindful of my safety during the exam. ______

Comments: (comment specifically on your overall comfort level with the physician; would you come back to him/her)
