# Supplementary material for: The use of standardized patients for mock oral board exams in neurology: a pilot study
Source: BMC Med Educ. 2006 Apr 25;6:22. doi: 10.1186/1472-6920-6-22 (PMC1464094; doi:10.1186/1472-6920-6-22)
Supplement: Additional file 3 — Appendix 3-Case Scenario for "Phyllis Jones" (PGY 4) [file 1472-6920-6-22-S3.doc]

# Appendix 3—Case Scenario for “Phyllis Jones” (PGY 4)

**Case Summary:**

You are a 33-year-old woman who has come to the neurology clinic on a referral from your primary care physician. You have been having some neurological problem that you need diagnosed and managed.

# History of Your Problems

You first noticed some problems about a year ago. One day you experienced numbness and tingling in your right leg and groin. It came on gradually over a three-hour period.

The numbness was in your whole leg and was accompanied by pain in your lower back. You also had a sensation of a “rush of heat” when you flexed your neck or back. If asked, you did NOT feel any weakness in your leg or loss of control of your bowel or bladder. Over the next 2-3 days the symptoms gradually went away.

You did not suffer any injury or have any time of illness before this episode. It was annoying, but you did not go to a doctor at that time. Since then, whenever you have tried to jog, you have had mild recurrence of the same symptoms. The symptoms seem to start as soon as you get warmed up a little. It seems to take less time in hotter weather. When you stop and rest; they go away.

Over the past 4 or 5 days you have been experiencing constant lower back pain with tingling and numbness in your right leg and groin area like before. You would describe the pain as a 6 on a 1-10 scale, if asked. Both legs feel weak and you’ve had some trouble walking. You’ve stumbled a few times when walking, but have NOT fallen down. During the first 2 days of this recent episode your balance was off. When you would stand your knee would tremble and jerk making you very unsteady. During those first two days you also had a couple of occurrences of bladder incontinence (wetting yourself). The bladder and balance problems seem to be better the last 2 days, but the pain is still there.

Your primary care doctor gave you some steroids (Prednisone). You starting taking it the day you saw the doctor. The first day you took 60 mg; yesterday you took 50 mg and today you are supposed to take 40 mg. You don’t think the steroids have helped much.

If asked about vision, you say that you have had some blurry vision in your left eye about 6 months ago. If asked, it didn’t really hurt, but there did seem to be some pressure behind your eyeball. Your vision has gotten better, but is still not back to normal. You’ve been avoiding going to the eye doctor.

# Past Medical History

You were generally very healthy until all of this started. You take no medications regularly (except those already discussed). You are allergic to penicillin. If asked, you break out in a head to toe rash if you take penicillin.

# Family History

Your father has diabetes. He takes pills for it; you’re not sure which. Several other relatives on dad’s side have diabetes, as well.

Your mother died of ovarian cancer about 4 years ago. Your mother’s sister had breast cancer. She had surgery and chemotherapy and has survived about 6 year since.

Your mother also suffered from arthritis.

# Social History

You work as a landscape designer. It has been impossible to work the last 4-5 days because of your symptoms.

You’ve been married for 6 years. You do not have any children.

You have never smoked. You have a drink “very little”. If asked, you have one drink per week at most. You have never used any other drugs.

# Review of Systems

If asked **directly** about the following; for the past 2 months

1. You are extremely tired lately.
2. You’ve been somewhat depressed. You’re not really sure why. You just don’t seem to enjoy things as much as before.
3. You’ve had a hard time falling asleep.
4. Your appetite is decreased.

# Physical Exam

During the entire encounter you have a flat affect. You have decreased visual acuity in your left eye (just do your best on eye chart testing with the right eye, but get a few wrong in the 20/50 or 20/60 line with the left—if they ask you to go to smaller numbers from there, just say you really can’t see it well and you’d just be guessing). You have mild weakness and spasticity in both legs. Remember that consistency is key—the legs will be weak during the entire history and exam (don’t fidget during history or move your legs easily).

Remember for weakness that you should not “overplay” weakness; it should be subtle despite this patient’s “best effort”. Try to keep 60-80% of your power present at all times in the legs, and when they pull against you keep consistent effort (do not jerk or “ratchet” if possible). Do not produce “weakness” by contracting other muscles not being tested—just focus on reduced power for the movement in question and relax all other muscles

Your reflexes are somewhat exaggerated in your legs, but normal in your arms. You have brisk or faster reflex responses in both legs as compared to the arms. Remember that it is the speed of the muscle twitch that counts and DO NOT exaggerate the movement caused by the reflex (does not look real).

You have some decreased sensation in your legs. If asked to compare to the normal sensation in your arms, you can say that it is 50% in the legs, maybe less on the right compared to the left (25% vs. 50%).

Both big toes go up if they scratch the bottom of your foot, side of your foot, if they run their knuckles down the front of your leg, or if they stick a pin into the top of your big toe. The up going movement should be very subtle and if you have a hard time, it is better for it to not move (often seen) as compared to an exaggerated upward movement (doesn’t look real).

# Phyllis Jones SP Checklist

**Resident Name _________________________________________________**

**SP Name ___________________________________________________**

The doctor discovered that:

1. I am 33 years old. ______

2. I am right/left handed. ______

3. I am here on a referral from my primary care doctor. ______

4. About a year ago, I had a some numbness in my right leg and groin. ______

5. The numbness came on gradually and went away gradually. ______

6. I noticed a “rush of heat” down my spine when I flexed my neck forward. ______

7. I did NOT lose control of my bladder during this first episode. ______

8. I have had recurrence of the same symptoms whenever I get hot (jogging). ______

9. The symptoms go away with rest. ______

10. For the past few days I have had constant numbness in my right leg and groin. ______

11. I have stumbled several times, but have NOT fallen down. ______

12. In the first two days my balance was “off”. ______

13. In the first 2 days I had several episodes of incontinence. ______

14. My balance has been better the last 2 days. ______

15. I have NOT experienced incontinence in the last 2 days. ______

15. I had some blurry vision in my left eye 6 months ago. ______

16. I take no medications regularly. ` ______

17. I break out in a rash if I take penicillin. ______

18. My father has diabetes. ______

19. I have not been able to work for the last 4-5 days. ______

20. I have never smoked ______

21. I drink no more than 1-2 drinks per week. ______

22. I have felt more “depressed” than usual lately. ______

23. I have been very tired lately. ______

24. I understood the doctor clearly during the history and exam. ______

25. The doctor used words that I could understand. ______

26. The doctor treated me with respect. ______

27. The doctor was mindful of my safety during the exam. ______

Comments: (comment specifically on your overall comfort level with the physician; would you come back to him/her)
