# Supplementary material for: The use of standardized patients for mock oral board exams in neurology: a pilot study
Source: BMC Med Educ. 2006 Apr 25;6:22. doi: 10.1186/1472-6920-6-22 (PMC1464094; doi:10.1186/1472-6920-6-22)
Supplement: Additional file 4 — Appendix 4-Faculty Evaluation Form [file 1472-6920-6-22-S4.doc]

**Appendix 4**

**Faculty Evaluation Form**

**Neurology Resident Exam Attending Physician Checklist**

**Patient: Peggy Cusick**

Resident Name _________________________________________________

Evaluator Name _________________________________________________

**Please score on a 1-3 scale. 3=adequate 2=borderline 1=inadequate**

The Resident:

1. Asked the patient’s age. 3 2 1

2. Asked the patient’s handedness. 3 2 1

3. Asked the chief complaint. 3 2 1

4. Asked about the history of the present illness/ 3 2 1

neurology symptoms

5. Determined the time course of the symptoms. 3 2 1

6. Obtained the past medical history. 3 2 1

7. Obtained a list of medications. 3 2 1

8. Obtained a history of allergies. 3 2 1

9. Obtained a family history. 3 2 1

10. Obtained a social history. 3 2 1

11. Performed a focused review of systems. 3 2 1

12. Washed his/her hands. 3 2 1

13. Took vital signs. 3 2 1

14. Performed the relevant portions of the general exam. 3 2 1

15. Performed a mental status exam. 3 2 1

16. Performed a cranial nerve exam. 3 2 1

17. Performed a motor exam. 3 2 1

18. Performed a sensory exam. 3 2 1

19. Performed a coordination exam. 3 2 1

20. Performed gait testing. 3 2 1

21. Tested deep tendon reflexes. 3 2 1

22. Tested plantar responses. 3 2 1

23. Was respectful and compassionate with the patient. 3 2 1

24. Communicated effectively with the patient. 3 2 1

25. Was mindful of the patient’s safety. 3 2 1

Comments: (Specifically comment on whether the resident convinced you that he/she is safe to practice in the real world)
