# Supplementary material for: The use of standardized patients for mock oral board exams in neurology: a pilot study
Source: BMC Med Educ. 2006 Apr 25;6:22. doi: 10.1186/1472-6920-6-22 (PMC1464094; doi:10.1186/1472-6920-6-22)
Supplement: Additional file 5 — Appendix 5-Breaking Bad News Exercise-"Peggy Cusick" SP Checklist [file 1472-6920-6-22-S5.doc]

# Appendix 5

# Breaking Bad News Exercise—Peggy Cusick

# SP Checklist

The doctor:

1. Used words that I understood. ______

2. Explained the diagnosis and implications clearly. ______

3. Was empathetic and compassionate. ______

4. Had body language that was appropriate for the situation. ______

5. Appeared nervous. ______

6. Repeated the diagnosis more than once to be sure it registered with me. ______

Comments:

# Faculty Checklist

The doctor:

1. Used words that the patient understood. 3 2 1

2. Explained the diagnosis and implications clearly. 3 2 1

3. Was empathetic and compassionate. 3 2 1

4. Had body language that was appropriate for the situation. 3 2 1

5. Appeared nervous. 3 2 1

6. Repeated the diagnosis more than once to be sure it registered 3 2 1

with the patient.

7. Accurately described the symptoms that the patient might. 3 2 1

encounter.

8. Accurately described the planned events/management that 3 2 1

the patient would undergo.

9. Did NOT leave the patient with a sense of abandonment (gave 3 2 1

her the sense that he/she would be there for her in the future.

Comments:
